# Social Stigma: The Hidden Threat of COVID-19

**DOI:** 10.3389/fpubh.2020.00429

**Published:** 2020-08-28

**Authors:** Ahmed Samir Abdelhafiz, Mohamed Alorabi

**Affiliations:** ^1^Department of Clinical Pathology, National Cancer Institute, Cairo University, Cairo, Egypt; ^2^Department of Clinical Oncology, Faculty of Medicine, Ain Shams University, Cairo, Egypt

**Keywords:** COVID-19, pandemic, spread, Egypt, social stigma

## Abstract

COVID-19 infection has been recognized as a pandemic by the World Health Organization. Efforts to prevent the spread of the disease are threatened by the appearance of disease-associated social stigma in society. In Egypt, a small wave of stigma directed at different groups started to appear. Here we report the features of COVID-19-associated stigma in Egypt and suggest recommendations to overcome this stigma before it grows and have physical and psychological impacts on society.

## Introduction

On the 11th of March 2020, the WHO declared the novel coronavirus disease (COVID-19) as a global pandemic (1). The disease rapidly spread through the world, affecting millions of people, with a mortality of about 5.7% (2). Since there is no approved vaccine or treatment for COVID-19, efforts to fight the disease focused on prevention of spread. These efforts included political decisions to apply social distancing from one side and public health education to increase awareness of the individuals about the disease and how to protect themselves on the other side (3).

However, the unprecedented situation of the current pandemic, where news spread instantly through media, could be associated with panic buying (4), fears, stereotyping, and the appearance of stigma directed at different groups in society. A survey was conducted on the general public in China to better understand the psychological impact, anxiety, depression, and stress during the initial stage of the COVID-19 outbreak. The authors found that 53.8% of respondents rated the psychological impact of the outbreak as moderate or severe; 16.5% reported moderate to severe depressive symptoms; 28.8% reported moderate to severe anxiety symptoms; and 8.1% reported moderate to severe stress levels (5). Four weeks in the peak of the COVID-19 pandemic, 34.8% of respondents reported stigma or discrimination by people from other countries (6). Furthermore, vulnerable groups like psychiatric patients are at higher risk of adverse mental health and faced more stigma during the COVID-19 pandemic (7). Healthcare workers also suffered from burnout and faced tremendous stress (8, 9) and potential stigma.

Here we report the current COVID-19 situation in Egypt, some features of the social stigma that emerged with the spread of COVID-19, and present our recommendations which can help limit this wave especially in Egypt.

## COVID-19 in Egypt

During the first week of March 2020, the Egyptian Ministry of Health and Population (MOHP) announced the first case of an Egyptian citizen infected with SARS-COV-2. Since then, a growing number of cases have been reported to reach about 64,000 cases and more than 2,700 deaths by the end of June (10). Although MOHP used different means of communication to educate the public about the disease, we reported a significantly lower level of knowledge among older, lower-income, less educated people and rural residents in our recently published survey about the knowledge, perceptions, and attitude of Egyptians toward COVID-19 (11). Most participants believed in the danger of the diseases, and about one quarter of them thought that infection is associated with stigma (11). Taken together, we think that although a certain degree of knowledge has been gained by some categories in the society, this has been associated with growth of rational and irrational fears toward the disease, its risk, and potential sources of infection.

## COVID-19-Associated Stigma

During outbreaks or pandemics, human fear arises from the anxiety about a disease of an unknown cause and possible fatal outcome, especially when infection control techniques such as quarantine and isolation are applied to protect the community (12, 13). In the past, stigma has been associated with different infectious diseases (4) and resulted in discrimination against these patient groups, which caused negative consequences both on the individuals and society (14). These features, which have also been reported during the COVID-19 pandemic in different studies, may result in stigmatization of the potentially infected that flourishes with dramatic stories in media and through the internet (5, 6).

During the current COVID-19 pandemic, several features of stigma have been reported worldwide mainly toward individuals from Asian descent, those with recent travel history, and healthcare professionals (15, 16).

There are several reports of xenophobia in Europe, USA, and many countries around the world directed mainly toward Asian foreigners (17–20). A single incident for discrimination against a person with apparently Asian features has been reported in Egypt. It was interesting that this incident was faced with rejection from society, including governmental officials (15).

The main religions in Egypt are Islam, followed by Christianity. Both religions have funeral rites that call for the burial of the body. Reports about locals refusing to bury the body of dead persons from COVID-19 have been published in Egypt as it might be a source of infection (21, 22). Similar reports have been published in Indonesia as well (17). The WHO declared that there is no evidence of possible spread of infection from dead bodies and released a guide on the safe management of a dead body from COVID-19 (23). Those incidents sounded the alarm for the wave of stigma, where the call for interference from the parties concerned is required.

Another dangerous feature of stigma is the one against healthcare professionals. In our study about the prevalence of burnout during the COVID-19 pandemic, more than one third of Egyptian doctors participating were found to suffer from burnout (data not published). Since healthcare workers come in the frontline among groups susceptible to infection, fears from communication with them have been reported in several forms in Egypt as well as other countries. For example, incidents have been reported where taxi drivers refused to drive medical doctors, restaurants refused to deliver food to hospitals, and residents refused to have healthcare professionals as neighbors (24–27). More than three quarters of Egyptian physicians participating in the aforementioned study about COVID-19-associated burnout believed that there is a stigma against health professionals and linked harassment by patients' families with different dimensions of burnout. A document by WHO about mental health and psychosocial considerations during the COVID-19 outbreak has pointed out to this type of stigma and provided recommendations to deal with it (28). Collectively, these individual incidents point to a potential hidden threat, which may be reflected in the form of underreporting of cases, fear to seek medical care, and a negative effect on the mental and emotional health of stigmatized groups (16).

## Discussion and Recommendations

Studies have shown that during serious disease outbreaks, when the general public requires immediate information, a subgroup of the population that is at potentially greater risk of experiencing fear, stigmatization, and discrimination will need special attention from public health professionals (29, 30). Several measures to deal with the mental and psychological stress and stigma during the COVID-19 response have been published by WHO, Centers for Disease Control and Prevention (CDC), and United Nations International Children's Fund (UNICEF) (16, 28, 31). Among these measures, we would like to highlight the following recommendations, which we think are best relevant to the situation in Egypt. It should be noted that these recommendations should be executed through collaboration between the government, international health organizations, private media sector, nongovernmental organizations (NGOs), and social influencers.

1. For media platforms: These platforms should try to increase awareness without increasing fear. They should also warn from negative behaviors and support stigmatized groups. Wording to describe patients or infected persons should be chosen properly. Accurate data and information should be carefully selected. Healthcare workers should be supported, and their work should be appreciated. It is also important to amplify positive and hopeful stories of people who recovered from the disease (16, 28, 31). Such news will limit the feeling that the disease is fatal and will increase the level of empathy with patients.
2. For individuals: We recommend that individuals should minimize exposure to news about COVID-19 (28). Prolonged exposure is associated with exaggerated fear and negative reactions. Moreover, social media and other communication methods can be a source of misinformation, which may increase the level of stress (11, 16, 28).
3. For healthcare workers: Avoidance by some members in the community can be disappointing. Getting support from family, colleagues, and managers can help healthcare workers overcome these feelings. Providing emotional support to affected people during different stages of isolation/treatment can help them overcome the psychological impact of stigma if present and give a positive example to the society (28). Professional psychological support should be available to all stigmatized individuals and groups, including healthcare workers.
4. Social influencers: Including religious leaders, should have a role through communicating messages that can help reduce stigma and support stigmatized groups (31). Such role can be very valuable if the persons and message were carefully chosen. Interestingly, the Grand Sheikh of Al-Azhar, a prestigious Sunni Islam title in Egypt, recently gave a speech warning against stigma associated with COVID-19 (32).
5. Workplace: Harassment and stigmatization at work can have a substantial adverse impact on physical and mental health, which may be reflected in the form of reduced productivity and increased staff turnover (33). Employers should follow general measures for creating a healthy workplace (33). The economic factor should be taken into consideration while dealing with COVID-19 patients. Psychological and financial support should be available for patients, and transparent policies about these issues should be communicated in advance. Staff education and speaking out against negative behaviors can help to avoid these behaviors in the future (16). The government should request employers to prevent and curb discrimination against confirmed or suspected cases of COVID-19 within the business (34).

## Data Availability Statement

The original contributions presented in the study are included in the article/supplementary material, further inquiries can be directed to the corresponding author/s.

## Author Contributions

AA conceptualized the idea and started the manuscript writing. MA then added his input and revised the manuscript. All authors contributed to the article and approved the submitted version.

## Conflict of Interest

The authors declare that the research was conducted in the absence of any commercial or financial relationships that could be construed as a potential conflict of interest.
